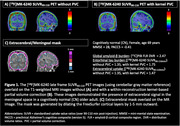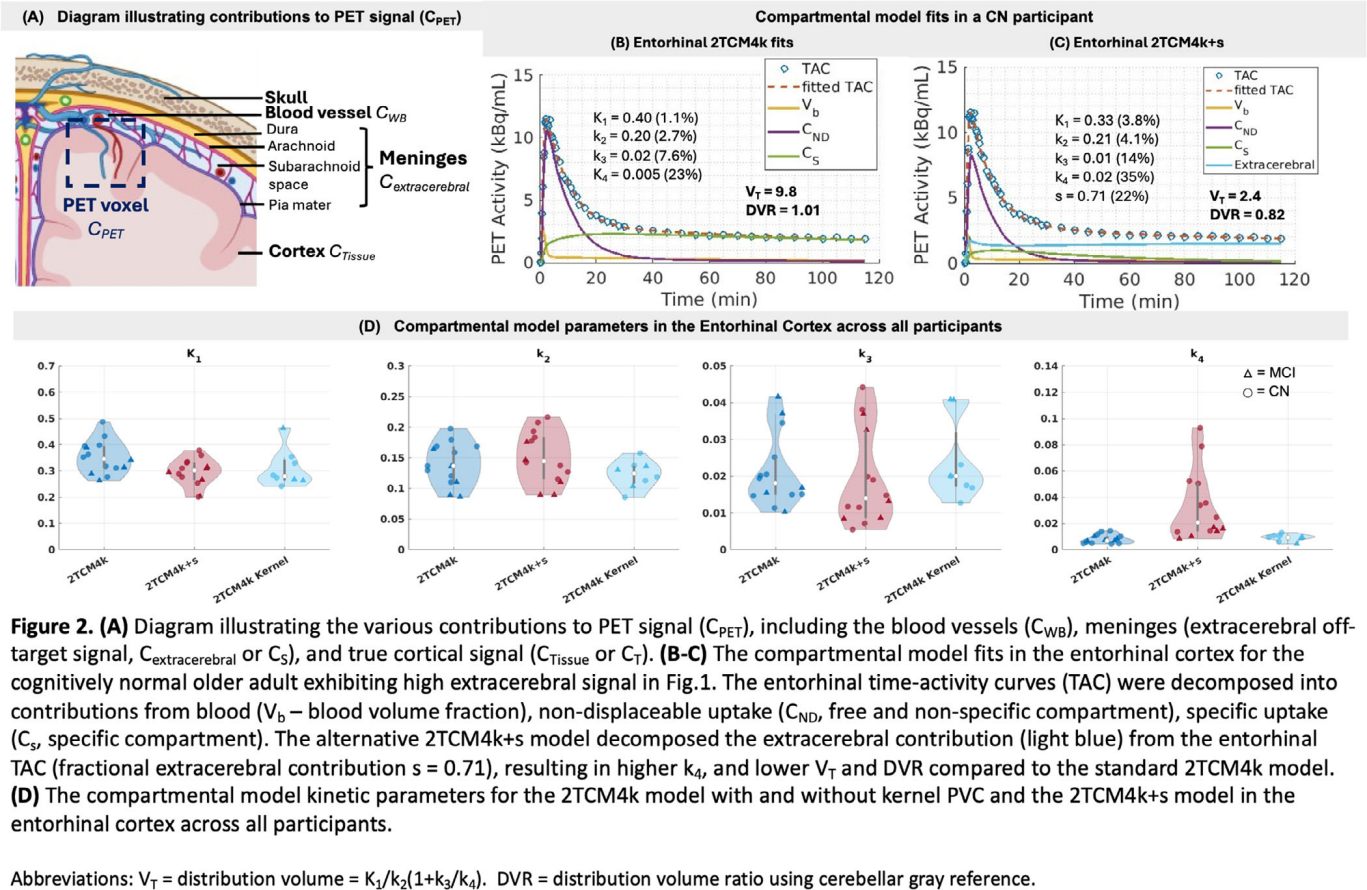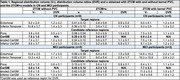# Quantifying extracerebral off‐target contamination in [^18^F]MK6240 PET imaging using compartmental modeling

**DOI:** 10.1002/alz70856_099911

**Published:** 2025-12-25

**Authors:** Jessie Fanglu Fu, Cristina Lois, Amal Tiss, Hasan Sari, Alex Becker, Justin S Sanchez, Nicolas J Guehl, Marc D Normandin, Georges El Fakhri, Keith A. Johnson, Julie C Price

**Affiliations:** ^1^ Massachusetts General Hospital, Harvard Medical School, Boston, MA, USA; ^2^ Yale School of Medicine, New Haven, CT, USA

## Abstract

**Background:**

[^18^F]MK6240 exhibits high affinity to Alzheimer's disease (AD) tauopathy, but meningeal or extracerebral off‐target signal (Figure 1AB) complicates early tau detection. We previously demonstrated that [^18^F]MK6240 off‐target signal follows irreversible kinetics, and an alternative compartmental model incorporating an extracerebral term (*s*) may quantify extracerebral contamination. Here, we further evaluate this model's performance and its impact on late‐frame SUVR compared to partial volume correction (PVC) for early tau detection.

**Methods:**

Thirteen participants (8 controls, CN; 5 mild cognitive impairment, MCI) underwent 2‐hr [^18^F]MK6240 PET, MRI, and arterial sampling (Table 1). A within‐reconstruction kernel‐based PVC using MRI information was applied (*n* = 8). Time‐activity curves were generated for extracerebral (Figure 1C), target, and reference regions. Regional uptake was modeled using: **1) 2TCM**, standard two‐tissue compartmental model, **2) 2TCM+s**, alternative model incorporating an additive extracerebral term, sC_s_ (*s* = fractional contribution of extracerebral signal *C_s_*) (Figure 2A), **3) 2TCM‐kernel**: 2TCM applied to kernel‐PVC data. Outcomes included distribution volume (V_T_), distribution volume ratio (DVR), and SUVR_90‐110min_. T‐tests (*p* <0.05) and Akaike Information Criteria (AIC) compared model parameters and performance. Cohen's d assessed effect size for CNs vs. MCIs using SUVR_90‐110min_ with/without PVC and with 2TCM4k+s correction.

**Results:**

The 2TCM+s model demonstrated the best AIC, followed by 2TCM‐kernel, then 2TCM. The extracerebral term *s* (0.16‐0.43, Table 1) reflected extracerebral contamination, being highest in regions near the meninges (e.g., entorhinal). Accounting for *s* led to lower V_T_ due to higher k_4_, particularly in highly contaminated regions (Figure 2B‐D). In early‐tau target regions, compared to DVR_2TCM_, DVR_2TCM+s_ was lower in CNs but similar in MCIs, while DVR_2TCM‐kernel_ was similar in CNs but higher in MCIs. The SUVR_90‐110min_ with 2TCM+s correction exhibited the largest effect size for differentiating CNs and MCIs, followed by kernel‐PVC SUVR_90‐110min_, with the smallest effect size in non‐PVC SUVR_90‐110min_ (entorhinal Cohen's d, non‐PVC: ‐0.58, kernel‐PVC: ‐1.0, 2TCM+s: ‐1.2).

**Conclusion:**

A compartmental modeling approach quantifies regional [^18^F]MK6240 extracerebral contamination, potentially improving V_T_, DVR, and SUVR accuracy and sensitivity for early tau detection, even compared to PVC data. Future studies will include more participants and extend these corrections to [^18^F]MK6240 data without dynamic acquisition, broadening applicability in clinical and research settings.